# Ultrasound Tissue Characterization of Vulnerable Atherosclerotic Plaque

**DOI:** 10.3390/ijms160510121

**Published:** 2015-05-05

**Authors:** Eugenio Picano, Marco Paterni

**Affiliations:** 1 Biomedicine Department, NU School of Medicine, Astana 010000, Kazakistan; 2 CNR (Consiglio Nazionale Ricerche), Institute of Clinical Physiology, 56124 Pisa, Italy; E-Mail: marco.paterni@ifc.cnr.it

**Keywords:** atherosclerosis, plaque, tissue characterization, ultrasound

## Abstract

A thrombotic occlusion of the vessel fed by ruptured coronary atherosclerotic plaque may result in unstable angina, myocardial infarction or death, whereas embolization from a plaque in carotid arteries may result in transient ischemic attack or stroke. The atherosclerotic plaque prone to such clinical events is termed high-risk or vulnerable plaque, and its identification in humans before it becomes symptomatic has been elusive to date. Ultrasonic tissue characterization of the atherosclerotic plaque is possible with different techniques—such as vascular, transesophageal, and intravascular ultrasound—on a variety of arterial segments, including carotid, aorta, and coronary districts. The image analysis can be based on visual, video-densitometric or radiofrequency methods and identifies three distinct textural patterns: hypo-echoic (corresponding to lipid- and hemorrhage-rich plaque), iso- or moderately hyper-echoic (fibrotic or fibro-fatty plaque), and markedly hyperechoic with shadowing (calcific plaque). Hypoechoic or dishomogeneous plaques, with spotty microcalcification and large plaque burden, with plaque neovascularization and surface irregularities by contrast-enhanced ultrasound, are more prone to clinical complications than hyperechoic, extensively calcified, homogeneous plaques with limited plaque burden, smooth luminal plaque surface and absence of neovascularization. Plaque ultrasound morphology is important, along with plaque geometry, in determining the atherosclerotic prognostic burden in the individual patient. New quantitative methods beyond backscatter (to include speed of sound, attenuation, strain, temperature, and high order statistics) are under development to evaluate vascular tissues. Although not yet ready for widespread clinical use, tissue characterization is listed by the American Society of Echocardiography roadmap to 2020 as one of the most promising fields of application in cardiovascular ultrasound imaging, offering unique opportunities for the early detection and treatment of atherosclerotic disease.

## 1. Tissue Characterization of Vulnerable Plaque: From Histology to Ultrasound

**Figure 1:**
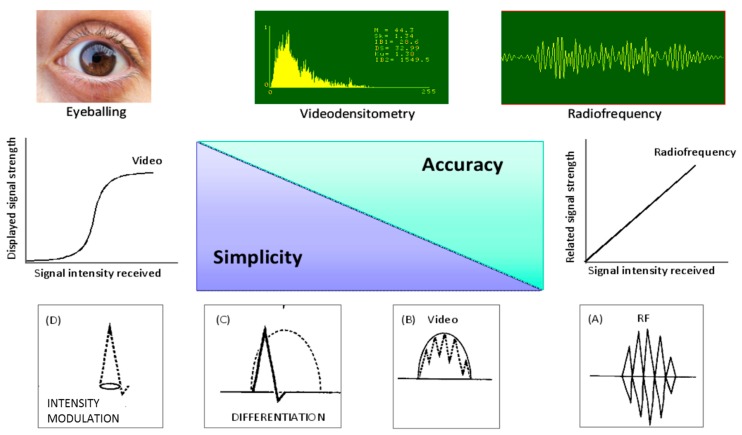
Approaches to tissue characterization: visual eyeballing analysis; videodensitometric analysis of digitized image by descriptors of image brightness and gray level spatial distribution; backscatter based sampling of received signal. The latter method is the most technically demanding, available in some but not all commercially available instruments, but it works on a linear relationship between received and displayed signal. This relationship is non-linear for visual and videodensitometric methods, working downstream to the electronic chain of signal processing, shown in the bottom panel, from right (panel **A**, original radiofrequency) to left (panels **B**–**D**, video, distorted, signal).

The underlying hypothesis in tissue characterization studies is that a different biochemical structure, internal architectural arrangement or physiologic state of normal *vs.* diseased tissue can affect the physical properties of the tissue and can therefore be detected by ultrasound. Tissue characterization can be performed using three main approaches with increasing degrees of complexity and accuracy: visual eyeballing, software-assisted videodensitometry of standard digitized images, and backscatter analysis of native radiofrequency signal (Figure 1). Visual eyeballing is the “first generation” approach (arising in the 1980s) and is still the only clinically viable option for large-scale use, but it can only detect the most obvious changes in tissue structure such as a hypoechoic, hyperechoic, or calcified carotid plaque. Videodensitometry is the “second-generation” approach, implemented since the mid-1990s, more objective than visual assessment and based on quantitative analysis of digitized video images. It samples the commercial video signal downstream to the processing chain distorting the linear relationship between received signal and displayed image. Radiofrequency analysis is a more technologically demanding “third generation” approach, commercially developed over the last 15 years and theoretically the most accurate, since the native ultrasonic signal is sampled upstream to the video display, and is not distorted by the post-processing function of the imaging chain. According to recent recommendations, “The long history of the ultrasound tissue characterization technique compared with its rare clinical use tells its own story in relation to its difficulty” [1]. This procedure is complex, subject to artifacts related to image settings, and the exact location of the sample volume. Calibrated backscatter has a value as a marker of fibrosis and calcification, but—the guidelines conclude—this methodology remains more of a research instrument than a clinical tool in echocardiography.

In spite of these recognized difficulties, the clinical yield of ultrasonic tissue characterization remains especially attractive in atherosclerosis, especially for the acoustic identification of vulnerable or high-risk plaques, a challenging but achievable target—as recently outlined by National Heart Lung and Blood Institute (NHLBI) Working Group [2]—for future research in the field. The carotid plaque is defined as “a focal structure that encroaches into the arterial lumen of at least 0.5 mm or 50% of the surrounding intima-media thickness or demonstrated a thickness of greater than or equal to 1.5 mm” [3]. For the clinician, there is a need to characterize “vulnerable plaque”, *i.e.*, the plaque susceptible to rupture, which can give rise to clinical complications, from embolization to thrombosis leading to symptoms, myocardial infarction, stroke and death. The vulnerability features are only weakly related to plaque size and stenosis and are also related to plaque morphology and histologic content: plaque size matters, but shape and content of the plaque also matter. Vulnerable, high-risk plaque is histologically different from stable, benign, clinically asymptomatic plaque—not only regarding its larger plaque burden but also for its higher content of lipids, with necrotic cores due to invasion of lipid pools by macrophages and other inflammatory cells with speckled micro-calcification (Table 1). The necrotic core can show hemorrhages due to extravasation of erythrocytes from the intimal neo-vascularization originating from the adventitia. The fibrous cap is usually thin, and the luminal contours may be irregular rather than smooth. All these histologic features can leave their readout on a variety of acoustic parameters, based on acoustic backscatter, attenuation, spatial texture, angular variability, plaque neo-vascularization detected through contrast administration, and acoustic internal homogeneity of spatial gray-level distribution. In order to have a comprehensive evaluation of plaque prognostic potential we need the assessment of plaque hemodynamic severity—as can be optimally provided by Duplex scan including Doppler and conventional B-mode—but also better insight into plaque content and morphology, as potentially provided by the tissue characterization approach [4].

**Table 1:** The vulnerable plaque read-out: from histology to ultrasound.

Histology	Ultrasound
Outward remodeling	Stenosis > 70%
Decreased Fibrous Tissue	Hypoechoic core
Increased Lipid-Hemorrhages	Hypoechoic core
More necrotic core	Dishomogeneous texture
Macrophages—inflammation	Dishomogeneous texture
Micro-calcification	Spotty hyper-dense foci
Endothelial rupture	Irregular border by CEUS
Intimal neovessel formation	Vascularization by CEUS

CEUS, contrast-enhanced ultrasound.

## 2. Tissue Characterization of the Atherosclerotic Plaque: *Ex Vivo* Studies

In the 1984 edition of Braunwald’s classic Textbook of Cardiology, Wissler stated that “at our present stage of technology and knowledge, it is virtually impossible to evaluate the quantities of the major components in any given plaque in the human subject at any specific time, short of surgical removal or examination at autopsy” [5]. Since then, there has been growing interest in characterization of the acoustic properties of the vascular wall to identify and define the composition of atherosclerotic plaque non-invasively, and several *in vitro* studies have established a solid experimental foundation of ultrasonic tissue characterization of the atherosclerotic plaque. These studies aimed (1) to clarify the biological determinants of vascular acoustic properties; (2) to test, under controlled conditions, ultrasonic parameters of potential diagnostic use; (3) to propose an anatomic-geometrical model of arterial scatterers in different stages of normality and disease; and (4) to orient the technological efforts necessary to translate the most meaningful laboratory information into a clinically feasible tool. 

The normal wall can be distinguished from atherosclerotic plaque by a variety of acoustic parameters. The peak amplitude value of reflected signal is low in normal walls and fatty plaques, intermediate in fibrous plaques, and highest in calcific plaques. This index is strongly phase-sensitive and angle-dependent, but also very simple [6]. Plaques can also be identified with parameters based on acoustic attenuation [7]. In particular, calcific plaques show an attenuation 700% greater than that found in normal wall, and 300% greater than that found in atherosclerotic non-calcific plaques. This finding is the experimental counterpart of the clinical echo finding of acoustic shadowing associated with focal calcification. A third parameter of interest for characterizing atherosclerotic plaque as well as in the elaboration of an anatomic-geometrical model of arterial scatterers is the backscatter angular dependence [8]. In contrast to the echoes arising from the myocardium, which are relatively independent of the angle of incidence of the ultrasonic beam to the tissue, those arising from arterial walls are generally said to be of a specular type. This is a major limitation to any application based on a quantitative diagnostic approach *in vivo* because specular reflectors give rise to a signal whose amplitude is highly dependent on the angle of incidence of the ultrasonic beam to the tissue target. The backscatter coefficient, measured at the single frequency of 10 MHz, was evaluated at a normal angle of incidence of the interrogating beam to the tissue sample and over an angular span of 60° (±30° around normal incidence). Angular scattering measurements identified a directive and a non-directive pattern. The directive pattern was characterized by a strongly angle-dependent backscatter that falls abruptly when the beam is moved slightly away from normal incidence. This pattern was typical of calcific, fibrous, and to a lesser extent, fibrofatty and normal samples. The non-directive pattern is characterized by a backscatter that is not significantly angle-dependent and fluctuates throughout the entire angular range. This was typical of fatty samples.

The histological architecture and biochemical composition of the arterial wall might be a reasonable morphological substrate for the recorded difference in angular scattering, which is determined by size and orientation of the scatterers relative to the ultrasonic beam. A directive angular response may be attributable to simple planar organization of the targets within the tissue. Scatterers in the normal wall might be physically identified in the thin elastic membrane present within the normal media layer and oriented perpendicularly to the beam axis. They give rise to directional scattering typical of that in structures in which large plane interfaces exist within the scattering volume. In fibrous and calcified specimens, the scatterers might be physically identified in thick collagen bundles and calcium laminae, which like elastic membranes are oriented perpendicularly to the beam. This might explain the very high directivity of these plaques. In fatty plaques, lipids accumulate in the intima, mainly in the amorphous state but also as cholesterol crystals. Such crystals are comparable in size to the wavelength of the beam, and are spatially arranged in a random fashion. The absence of a spatial orientation and the small size of the scatterers both contribute to the nondirective type of angular scattering in the plaques. In the fibrofatty plaque, the markedly directive response is probably attributable to the fibrous cap; however, the coexistence within the scattering volume of a nondirective structure (the fatty core, absent in the purely fibrous plaques) partially blunts the directivity of the angular response, which is substantially less than in the fibrous samples.

Another potentially useful parameter is the spatial distribution of echo density in an arterial region of interest. In this approach, the information is less dependent on the absolute value of echodensity, and more related to the relative value of different pixels within a region of interest [9]. The shape of the integrated backscatter amplitude distribution is more spread out and flat in the atherosclerotic region.

Another approach is the analysis of the echo signal in time domain (across the depth wall), conceptually similar to the old A-mode representation of ultrasound [10]. If one measures only the first interface aqueous-intimal echo, there is a variable amplitude value for all plaque subsets except for lipidic plaque, which shows a consistently low amplitude value.

These findings were confirmed by different laboratories using qualitative assessment of B-mode images [11] or more quantitative backscatter analysis [12,13,14] and the overall conclusion is that the atherosclerotic plaque composition leaves several ultrasonic fingerprints which can be fruitfully used for tissue characterization of the vulnerable plaque (Table 1, right side). Even under ideal imaging conditions (*in vitro*, no interposed tissue, controlled angle of insonation, quantitative analysis of reflected signal) lipids and hemorrhages cannot be distinguished by ultrasound, and both appear as low echogenic (“soft”) tissue.

## 3. *In Vivo* Ultrasonic Tissue Characterization

Clinical studies have confirmed that the vulnerable, lipid-rich plaque can be identified in the carotid with all three approaches of tissue characterization: visual eyeballing [15,16,17,18,19], videodensitometry [20,21,22] and backscatter [23,24,25] (Table 2). Whatever the method, plaque morphology assessment is critically dependent on image quality and the echogenicity is usually normalized for an internal standard, such as—for visual assessment—the flowing blood (black) or far wall media-adventitia surface (white). Eyeballing characterization of plaque texture is subjective and operator-dependent, polluted by technological speckle, but it remains an attractive option since it is simple, straightforward, and still capable of detecting quickly and simply any obvious changes in plaque composition [15,16,17,18] with an acceptable reproducibility in controlled conditions when compared to more complex methods [19]. Videodensitometry is quantitative, and still widely applicable in the current era, with most instruments generating a picture describing the image texture through mean gray level and higher order statistics (such as entropy) for spatial distribution of texture.

**Table 2:** Ultrasonic tissue characterization: tools.

Parameter	B-Mode Ultrasound Imaging		
	Vascular	Transesophageal	Intravascular
Ultrasound frequency	5–15	5–10	15–20
Signal-to-noise ratio	++	++	+++
Accuracy	++	++	+++
Prognostic value	++	++	+++
Applicability	Bedside	Echo lab	Cath lab
Invasiveness	Non-invasive	Semi-invasive	Invasive
Main target artery	Carotid (femoral)	Thoracic Aorta	Coronary

− = poor; ± = fair; + = good; ++ = very good; +++ = excellent.

Videodensitometry is quantitative, and still widely applicable in the current era, with most instruments generating a picture describing the image texture through mean gray level and higher order statistics (such as entropy) for spatial distribution of texture. The vulnerable plaque is more often hypoechoic, with lower mean gray level and higher entropy values (an index of spatial heterogeneity of gray level distribution) in the region of interest [19,20,21,22]. Calibrating the system in a 256 gray level scale with blood equal to 0 (=black), perfect white = 255 and the far wall, strongly reflective adventitial interface = 190, the lipid-hemorrhagic plaques (with blood-like backscatter) remain below 30, with stable plaques showing higher mean gray levels and—with texture analysis—higher entropy values associated with less homogeneous spatial pattern. Radiofrequency analysis is more technologically demanding, and theoretically more accurate, since the “native” ultrasonic signal is sampled, which is not distorted by the post-processing function of the imaging chain [23,24,25]. It can be applied in the carotid and in the coronary [24,26] districts, with similar findings. Calibrating the system with blood equal to 0 decibels (dB) and the perfect artificial reflector equal to 50 dB, the lipid-hemorrhagic plaques (with blood-like backscatter) remain below 14, fibrous and fibro-fatty plaques in between 14 and 26, and calcific plaques above 27. Although the radiofrequency approach is the most quantitative, the other, simpler approaches can also provide clinically valuable information for *in vivo* characterization of the ultrasonic plaque. The echogenicity is usually expressed with a qualitative score with visual assessment (from black = 1 to white = 4), in grey level units with videodensitometry (from black = 0 to white = 255), in absolute decibel values in radiofrequency analysis (Table 3). Additional echographic features of plaque instability are the neovascularization of the plaque and the irregular contour of plaque surface which are best detected by contrast-enhanced ultrasound (CEUS) [27,28,29].

## 4. Ultrasound Plaque Morphology as an Index of Clinical Instability

Although the field suffers from lack of standardization, steady changes in image technology, and lack of prospective randomized trials, over the last 30 years a series of observational studies has built up respectable evidence that an unstable plaque morphology by ultrasound identifies a higher risk subset when evaluated by different approaches (visual, videodensitometry, backscatter, CEUS) in different districts (carotid, aorta, coronary arteries) with different methods (vascular, transesophageal, intravascular ultrasound), on different populations (from acute and stable coronary patients to stable and unstable cerebrovascular or peripheral artery disease patients to asymptomatic persons at risk).

Subjects with echo-lucent and/or heterogeneous and/or neovascularized (by CEUS) atherosclerotic plaques in carotid arteries have increased risk of ischemic cerebrovascular and cardiovascular events independently of both degree of stenosis and cardiovascular risk factors [30,31,32,33,34,35,36,37].

The link between echo plaque structure and prognosis do not appear to be limited to the carotid arteries but may apply to virtually all vascular districts where atherosclerotic plaques can be imaged by ultrasound technology including femoral artery [38]. In the ascending thoracic aorta, non-calcified aortic plaques detected by TEE in brain infarction have been associated with a tenfold increased risk of subsequent events when compared to calcified plaque [39].

In coronary arteries evaluated by intracoronary ultrasound, a meta-analysis of 16 studies totaling 1693 patients who underwent PCI showed that the necrotic core (hypoechoic) component derived from virtual histology—IVUS at the minimum lumen sizes were significantly greater in the embolization (no-reflow) group compared with the no-embolization group [40]. Necrotic core was identified in the color-coded analysis as “red” (different from green, fibrotic, yellow-green, fibrofatty, and white, dense calcium). The larger the amount of attenuated coronary plaque by intracoronary ultrasound, the greater the likelihood of no-reflow [41].

Recent studies support the concept that plaque instability is not merely a local vascular incident but rather that plaque instability exists simultaneously at multiple sites of the vascular bed [42]. Similar features can be recognized in unstable patients not only in the symptomatic carotid plaque but also in the contralateral asymptomatic side, suggesting that the vulnerable plaque is also a part of the systemic inflammatory process of the vulnerable patient [43]. Ultrasonic features of instability are potentially a biomarker not only of the vulnerable plaque but—in a certain sense—of the vulnerable patient.

## 5. Clinical Implications

Information on plaque characterization can be obtained by ultrasound and is clinically important for several reasons. First, ultrasonically heterogeneous or soft plaques are associated with lipids and hemorrhages and have a greater tendency to ulceration, embolization, and development of symptoms. Second, anti-atherosclerotic statin treatment may induce a biochemical remodeling of the atherosclerotic plaque, with greater effect on lipidic components than on overall plaque size, which appear more echo-dense (and therefore less vulnerable) after therapy both in the coronary [44,45] and the carotid [46,47,48,49,50,51] arteries.

**Table 3:** Plaque imaging by ultrasound: criteria of instability.

Type of Plaque	Unstable	Stable
Visual assessment	Hypo-, Anechoic	Iso-, Hyper-echoic
	Heterogeneous	Homogeneous
	Irregular surface	Regular surface
Videodensitometry	Low median gray level	High median gray level
	High entropy	Low entropy
Radiofrequency	<13 dB	14–33 dB
CEUS	Neovessel Present	Neovessel Absent

Higher values correspond to higher echodensity. Visual assessment of echogenicity refers black as blood and white as far-wall adventitia interface (Gray-Weale, 1988 [16]). Homogeneity is defined according to Joakimsen, 1997 [17] and surface regularity as in Ibrahimi, 2014 [43]. Videodensitometry values are expressed in median grey levels (MGL, 0 black–255 white), with 0 = black as blood and 190 = bright as far-wall adventitia (Ibrahimi, 2014 [43]). Backscatter values are expressed in decibels (dB, calibrated with 0 dB = blood and 50 dB = stainless steel specular interface), according to Kawasaky, 2001 [24]. CEUS binary criteria for intimal neovascularization were proposed by Coli, 2008 [27].

Whatever the method available (visual, videodensitometry or backscatter-based), a simple description of plaque morphology can be helpful to the clinician as a clue to separate stable and unstable plaques (Table 4). Conventional echography and ultrasonic tissue characterization are not mutually exclusive, and any commercial device has—or will soon have—both conventional imaging and quantitative tissue characterization imaging built into the same hybrid basic hardware. Information on tissue characterization of the atherosclerotic plaque can be obtained with other imaging techniques, including MRI, CCTA, PET with FDG and—invasively—by OCT [4].

**Table 4:** Ultrasound appearance and plaque risk.

Risk	Low-Risk	High-Risk
Plaque border profile	Smooth	Irregular
Echo-density	Iso-, Hyper-echoic	Hypo-, Anechoic
Plaque luminal border *	Regular	Irregular
Plaque neovascularization *	Absent	Present
Spotty calcification	Rare	Frequent
Massive calcification	Frequent	Rare
Plaque burden	Low (<40% stenosis)	High (>70% stenosis)

* By CEUS, contrast-enhanced ultrasound. Carotid plaques are imaged by Duplex scan, coronary plaques by invasive intracoronary ultrasound.

The ultrasound approach has clear advantages over other clinically viable imaging approaches used to detect the vulnerable plaque: non-invasive (differently from OCT—although intracoronary ultrasound can be used, with higher frequencies and better signal-to-noise ratio than vascular ultrasound), radiation-free (differently from CCTA and PET), low cost and high spatial and temporal resolution [4]. Carotid duplex imaging is suitable for evaluation of extracranial cerebral vessels, but cannot image intracranial portion of the carotid artery and is less accurate in presence of dense calcification [3].

## 6. Conclusions

Ultrasonic tissue characterization of atherosclerotic plaque began 30 years ago, and while not yet ready for clinical use it is today regarded as one of the most promising fields of application in cardiovascular ultrasound imaging. In particular, it has the potential to identify—for any given stenosis—vulnerable, lipid-rich, unstable plaques more prone to complications such as embolization and rupture, and also receiving the greatest benefit from pharmacological and mechanical intervention strategies to prevent such events. Although fascinating, the imaging approach has inherent limitations, since not all ruptured plaques have histologic features of vulnerability (and 20% have none of them), and not all vulnerable plaques by histology criteria do eventually rupture in their natural history [3]. With these caveats, the imaging of plaque vulnerability remains a reasonable approach to bridging the current gap in understanding clinical manifestations of atherosclerotic disease, especially regarding the striking clinical mismatch between atherosclerosis extent and severity and its clinical manifestations, and the therapeutic mismatch between reductions of clinical events obtained with statins and the limited—if any—reduction in atheroma size. New quantitative methods beyond backscatter (to include speed of sound, attenuation, strain, temperature, and high order statistics) will be developed to evaluate vascular tissues. These image methods may offer opportunities for the early detection and treatment of the disease [52]. Once the methodology and analysis have been standardized, the stage will be set for future prospective randomized trials to evaluate whether quantitative tissue characterization-based information on plaque vulnerability can be used to tailor risk and treatment in patients with clinically symptomatic and high-risk asymptomatic atherosclerosis.
